# Comparative efficacy of intrathecal morphine and adductor canal block in the knee arthroplasty population: a retrospective multi-centre cohort study

**DOI:** 10.1186/s12871-024-02740-1

**Published:** 2024-10-10

**Authors:** Brigid Brown, Tim Soon Cheok, David Worsley, Hidde M. Kroon, Nathan Davis, Ruurd L. Jaarsma, Job Doornberg, D-Yin Lin

**Affiliations:** 1 Department of Anesthesiology, Flinders Medical Centre, Flinders Drive, Bedford Park Adelaide, South Australia 5042 Australia; 2 College of Medicine and Public Health, Flinders University, Adelaide, South Australia Australia; 3 Department of Orthopedic and Trauma Surgery, Flinders Medical Centre, Adelaide, South Australia Australia; 4 Department of Surgery, Royal Adelaide Hospital, Adelaide, South Australia Australia; 5 Adelaide Medical School, Faculty of Health and Medical Sciences, University of Adelaide, Adelaide, South Australia Australia; 6 Department of Orthopedic Surgery, University Medical Centre Groningen, Groningen, The Netherlands

**Keywords:** Anesthesiology, Regional, Adductor canal, Morphine, Intrathecal, Analgesia

## Abstract

**Background:**

Finding the balance of good postoperative analgesia while facilitiating mobility is important for a safe and satisfactory patient experience during Total Knee Arthroplasty (TKA). This study aimed to compare the efficacy of intrathecal morphine, adductor canal block, and their combination in optimizing pain management and postoperative recovery in TKA patients. This retrospective analysis of prospectively collected data evaluated postoperative pain scores, time to mobilisation, and length of hospital stay.

**Methods:**

1006 consecutive patients undergoing elective TKA across two large tertiary centres were included over six years. They were divided into one of four groups according to the type of analgesia received: Group N patients received no neuraxial morphine or regional block. Group B patients received adductor canal block (ACB) only. Group M patients received intrathecal morphine (ITM) but no regional block. Group BM patients received both ACB and ITM.

**Results:**

Patients who received an ACB had faster postoperative mobilization compared to those without (*p* < 0.001). Patients in Group BM had the lowest pain scores at rest (Visual Analogue Scale (VAS) 2.9) and with movement (VAS 5.3), while Group B patients experienced the highest pain scores at rest (VAS 3.7) and on movement (VAS 6.5) (*p* = 0.005). Patients who received ITM had the lowest opioid requirements (*p* < 0.001). There was no significant differences between groups in requirement for rescue pain management strategies (*p* = 0.06).

**Conclusions:**

The combination of ITM and ACB in patients undergoing TKA provides improved postoperative analgesia with lower postoperative opioid requirement and earlier mobilization compared with ACB or ITM alone.

## Introduction

Total knee arthroplasty (TKA) is an increasingly common orthopaedic surgery performed to improve pain and function in patients with degenerative knee conditions such as osteoarthritis [1].

With improvements in surgical techniques over the recent years, flow pathways have been developed with a focus on early mobilisation and physiotherapy as well as reduced length of hospital stay. The choice of anaesthetic technique has been impacted by these trends as TKA is associated with significant postoperative pain and opioid use [2]. Inadequate pain management can negatively impact the patient’s postoperative course by slower mobilisation and longer hospital stay [3].

Several anesthesia techniques have been employed to improve analgesia, such as neuraxial anesthesia with local anaesthetic and intrathecal opioids including morphine and fentanyl. Regional anesthesia has also become increasingly popular with the improvement in ultrasound technology and the evolution of fascial plane and motor sparing regional blocks [4]. Motor sparing regional techniques and shorter acting spinals have become popular to facilitate the requirements of early mobilisation and reducing length of hospital stay. Current procedure-specific postoperative pain management (PROSPECT) guidelines for TKA advocate for anaesthetists to provide a single shot adductor canal block for postoperative analgesia [5].

There is concern that this shift towards less invasive interventions such as motor sparing blocks may come at the expense of patient comfort and satisfaction in their perioperative journey [6]. Finding the combination of good postoperative analgesia while aiding patient movement by preventing muscle weakness is paramount for a safe and satisfactory patient experience [7]. This retrospective study aimed to evaluate different anaesthetic techniques in patients undergoing TKA to assess postoperative pain scores, time to mobilisation, and length of hospital stay.

## Patients and methods

We conducted a multi-centre retrospective cohort study using the Southern Adelaide Local Health Network Acute Pain Service database. This database contains prospectively collected data on all patients referred to the Acute Pain Service (APS) in two tertiary-level teaching hospitals in Australia (Flinders Medical Centre and Noarlunga Health Service, both in South Australia). Both hospitals provide a comprehensive orthopaedic service, which includes knee arthroplasty. The same group of orthopaedic surgeons and anaesthesiologists work across the two hospitals, and local rehabilitation protocols are the same.

Since 2017 there has been a significant uptake in ultrasound-guided motor sparing regional anesthesia techniques as part of the anaesthetic for TKA [8]. Multi-centre ethical approval was obtained from the South Australia Health Human Research Ethics Committee prior to this analysis being conducted (Approval No: 172.22). Informed written consent was obtained as part of surgical consent for the patients’ procedures, and all participants were re-consented verbally upon initial introduction to the Acute Pain Service team postoperatively.

### Patient selection

All patients undergoing TKA between 1st of January 2017 and 31st of December 2022 who were referred to the APS were considered for inclusion. These patients were then screened for the type of anesthesia administered and included in the study if they fit one of the four following groups: Group N consisted of patients who received no neuraxial morphine or regional block, Group B were patients who received adductor canal block (ACB) only, Group M were patients who received intrathecal morphine (ITM) but no regional block, and Group BM were patients who received both ACB and ITM. Exclusion criteria was: patients who received any other combination or types of blocks were excluded from the study, as were any patients who declined to form part of the Acute Pain Service database, or declined referral to or did not engage with the APS upon referral.

### Outcome measures and statistical analysis

Primary outcomes were time to mobilisation, requirement for rescue pain management strategies (including epidural anesthesia, patient-controlled analgesia, continuous analgesia infusion, and additional regional anesthesia), postoperative pain levels on movement and at rest using the Visual Analogue Scale (VAS) as assessed by the APS team on the ward, and postoperative opioid analgesic requirements. Rescue analgesia was given if found to be warranted by the APS review- the criteria for which was moderate to severe pain unresponsive or not adequately responsive to standard tablet based or subcutaneous analgesia. Standard analgesia was multimodal paracetamol, non-steroidal anti-inflammatories if no contraindication, and tramadol or oxycodone or fentanyl subcutaneously as needed.

Secondary outcomes were presence of opioid-related side effects including pruritis and nausea and vomiting, number of days of APS review required, and length of hospital stay. Pruritis was assessed using the Itch Man Score (IMS) on a scale from 0 to 3 (no itch, mild, moderate, and severe) while PONV was assessed on a four-point scoring system from 0 to 3 (none is scored as zero, mild/not requiring treatment is 1, moderate is 2, severe/persistent despite treatment is 3).

Baseline demographic data were recorded and analysed for each group. Categorical variables are displayed as frequency (percentages) and were compared using the Chi-Squared test. Continuous variables are displayed as mean (standard deviation). As the continuous data in our study did not meet the assumption of normality following the Shapiro-Wilk test, overall comparison of continuous variables was provided using the Kruskal-Wallis test. If there was significant difference between groups, pairwise analysis was then performed using the Mann-Whitney test. All statistical analyses were performed using STATA Version 18.0 (StataCorp, Texas, USA). The threshold for statistical significance was set at 95%.

## Results

From the APS database, 1,006 patients met the inclusion criteria. Breakdown into groups can be found in Table 1. Baseline demographics were comparable across all groups. Groups N and BM underwent fewer revision surgeries compared to the other two groups. All patients in Group M and BM received spinal anesthesia, whereas patients in Group N and B received a mixture of spinal and general anesthesia (Table 1).

**Table 1:** Baseline demographics and perioperative data

	Group *N* (No Block) *N* = 108	Group B (ACB Only) *N* = 423	Group M (ITM Only) *N* = 213	Group BM (ITM + ACB) *N* = 262	*P*-value
Age in Years, mean (SD)	66.9 (10.8)	69.7 (8.7)	69.3 (9.6)	68.5 (9.6)	0.14
Gender, n (%)					0.80
Female	61 (57.0)	260 (62.2)	130 (61.0)	160 (61.8)	
Male	46 (43.0)	158 (37.8)	83 (39.0)	99 (38.2)	
Primary vs. Revision, n (%)					**0.005**
Primary	102 (94.44)	412 (97.40)	199 (93.43)	259 (98.85)	
Revision	6 (5.56)	11 (2.60)	14 (6.54)	3 (1.15)	
Anaesthesia Type, n (%)					**< 0.001**
General	4 (3.70)	30 (7.09)	0 (0)	0 (0)	
Spinal	103 (95.37)	393 (92.91)	213 (100)	262 (100)	
Not recorded	1 (0.91)	0 (0)	0 (0)	0 (0)	

### Primary outcome measures

Patients who received an adductor canal block (Groups B and BM) mobilised earlier postoperatively compared to patients without a block (Groups N and M). Group B mobilised earlier postoperatively compared to Group BM (1.3 vs. 1.4 days, *p* = 0.001) and both mobilised earlier than patients in Group N without a block (1.5 days, *p* < 0.001). Patients in Group BM had the lowest pain scores at both rest and with movement, while Group B patients experienced the highest pain scores at rest and on movement (*p* < 0.001 both for rest and with movement). Patients in Groups M and BM who received ITM had the lowest opioid requirements, compared with patients in Group N who had the highest opioid requirements (*p* < 0.001). There was no significant difference between groups in requirement for rescue pain management strategies (*p* = 0.06).

### Secondary outcome measures

Patients in Group N required the longest follow up with the APS (*p* < 0.001). Patients who received only an ACB (Group B) had the shortest length of hospital stay at 3.1 days while patients who received ITM only (Group M) had the longest length of stay at 3.6 days (*p* = 0.002). There was no difference in postoperative nausea and vomiting scores between groups (*p* = 0.29). Group N had a significantly higher mean pruritus score (0.1) than Group B (0.05) and Group BM (0.04) with p-values of 0.04 and 0.02, respectively. Of note, there was no reported respiratory depression in any patient who received ITM. Analysis of primary and secondary outcome measures, as well as pairwise comparison of significant continuous outcomes, are shown in Tables 2 and 3.

**Table 2:** Outcome measures

	Group *N* (No Block) *N* = 108	Group B (ACB Only) *N* = 423	Group M (ITM Only) *N* = 213	Group BM (ITM + ACB) *N* = 262	*P*-value
Primary Outcomes					
Time to Mobilisation in Days, mean (SD)	1.5 (0.6)	1.3 (1.1)	1.6 (0.7)	1.4 (1.3)	**< 0.001**
Rescue Pain Management Strategies, n (%)	1 (0.9)	12 (2.8)	2 (0.9)	1 (0.4)	0.06
Movement Pain on POD 1, mean (SD)	6.3 (2.2)	6.5 (2.5)	6.1 (2.8)	5.3 (2.9)	**0.005**
Opioid Requirement on POD 1 in OME, mean (SD)	95.9 (116.3)	76.2 (87.2)	21.2 (44.9)	29.7 (37.7)	**< 0.001**
Resting Pain on POD 1, mean (SD)	3.3 (2.3)	3.7 (2.4)	3.4 (2.6)	2.9 (2.6)	**0.005**
Secondary Outcomes					
Side Effects					
Pruritus Score, mean (SD)	0.1 (0.3)	0.05 (0.2)	0.1 (0.4)	0.08 (0.2)	0.05
PONV Score, mean (SD)	0.2 (0.7)	0.4 (0.7)	0.3 (0.7)	0.3 (0.7)	0.29
Days Reviewed, mean (SD)	1.2 (0.5)	1.1 (0.3)	1.0 (0.3)	1.1 (0.2)	**< 0.001**
Length of Stay in Days, mean (SD)	3.5 (2.5)	3.1 (2.2)	3.6 (2.8)	3.4 (3.9)	**0.003**

**Table 3:** Pairwise comparison of significant continuous outcomes

	Time to Mobilisation	Movement Pain	Opioid Requirement	Resting Pain	Pruritus Score	Days Reviewed	Length of Stay
Group 1 vs. 2	**< 0.001**	0.61	0.98	0.19	**0.03**	**0.01**	**0.005**
Group 1 vs. 3	0.07	0.85	0.20	0.97	0.55	**< 0.001**	0.77
Group 1 vs. 4	**< 0.001**	**0.03**	0.40	0.14	0.05	**< 0.001**	0.08
Group 2 vs. 3	**< 0.001**	0.79	**< 0.001**	0.28	0.10	**0.04**	**0.002**
Group 2 vs. 4	**0.001**	**< 0.001**	**< 0.001**	**< 0.001**	0.52	0.09	0.24
Group 3 vs. 4	**< 0.001**	0.08	**< 0.001**	0.22	0.08	0.59	0.07

ACB: Adductor Canal Block, ITM: Intrathecal Morphine, OME: Oral Morphine Equivalents, PONV: Postoperative Nausea/Vomiting, SD: Standard Deviation

Pain scores are all measured on the Visual Analogue Scale (VAS) from 0–10

### Power calculation

As this is a retrospective study, we did not perform a-priori sample size calculation. Post-hoc power calculation for each primary outcome investigated showed adequate power. These were 96% for time to mobilization, 78% for rescue pain management strategies, 98% for pain on POD1 movement, > 99% for opioid requirement on POD1, and 86% for POD1 resting pain levels.

## Discussion

This cohort study compared different types of analgesia in patients undergoing TKA. Overall, patients who received a combination of ITM and ACB (Group BM) exhibited optimal efficacy compared to the other groups. This group mobilised earlier, had reduced pain scores at both rest and with movement, and required less opioids postoperatively. Importantly, there was no difference in morphine related side effects such as nausea and vomiting or pruritis between groups. Nor did patients experience delayed respiratory depression on the postoperative ward.

The literature is inconclusive regarding the best anaesthetic and analgesic profile for TKA. Some studies demonstrate the superior analgesic effect of ITM compared with regional anesthesia, reflecting the findings in this study [7, 9–11]. The results of this study are similar to Biswas et al., and demonstrate a better postoperative analgesic profile and lower opioid requirement when using a combination of ITM and ACB [7]. This was also supported in a study comparing ITM to no ITM (but no ACB) which concluded ITM provided better early postoperative pain scores and improved mobility distance [12]. Others conclude there is similar analgesic effectiveness of adductor canal or femoral nerve block and intrathecal morphine [4, 5, 13–15] and between ITM and local infiltration [16–18]. Contrastingly, some studies describe an inferior effect of ITM compared with local anaesthetic with higher opioid requirements at 24 h after ITM [5, 19].

While the pain score findings were statistically significant, the clinical significance may be less meaningful. The mean VAS at rest in Group BM was 2.86 while in Group B it was 3.73 (*p* < 0.001). This difference is only 0.87, which falls below the minimally important clinical difference of 1.37 [20]. However, the effect of the reduced VAS is supported clinically by a reduction in overall postoperative opioid requirement, where Group BM required 29.7 morphine equivalents postoperatively, Group B required 76.1 (*p* < 0.001) and Group N 95.9 (*p* < 0.001). This may suggest some meaningful improvement of pain in Group BM. Further, while not statistically significant, there was a trend towards less need for rescue techniques for uncontrolled pain in patients with ITM. In Group B (ACB only) 12 patients required rescue analgesia compared with 1 patient in the Group BM. Biswas et al. also noted that ITM plus ACB had the lowest incidence of uncontrolled pain requiring a rescue technique [7].

The side effects associated with intrathecal morphine have been well recorded and are often cited as a reason to avoid it in arthroplasty surgery. Of particular concern is the risk of delayed respiratory depression, with a reported incidence of between 0.26 and 3% with morphine doses given ranging between 0.15 and 0.8mg [21]. Some guidelines recommend avoiding ITM due to the respiratory depression risk and the subsequent need for increased respiratory monitoring [4, 22]. While the dose of intrathecal morphine has traditionally been as high as 1.5 mg, in current practice, with doses below 150mcg, there is almost no risk of respiratory depression, which we also found in the current study [14].

The incidence of other morphine related side effects, such as pruritis and postoperative nausea, are also reported variably in the literature. Some studies have noted an increase in pruritis postoperatively in patients receiving ITM compared with a regional block [4, 5, 11, 13, 15, 17] while others found no difference [7, 9, 12, 14]. Interestingly, the group with the highest postoperative pruritis score in the current study was Group N, who received no ITM or nerve block. This may reflect the significantly higher use of postoperative parenteral opioids this group required.

## Limitations

Some limitations of this study must be addressed. This is a retrospective analysis of prospectively collected data and is limited to the accuracy of the data collected during the patient’s admission. The number of patients in each group is unequal which may reflect a skewed aspect to the study.

Although the database is robust and consistently completed there are several indices of interest which were not routinely collected. For instance, preoperative patient pain scores, depression and anxiety scores, and patient satisfaction with the anaesthetic treatment would all have been interesting to include to provide a deeper understanding and comparison.

Furthermore, the data was only collected while the patient is under the care of the APS. Given the average days reviewed in this study is 1.1 days, it is not possible to comment about the longer postoperative patient course.

## Conclusions

The combination of ITM and ACB in patients undergoing TKA provides improved postoperative analgesia with lower postoperative opioid requirement and earlier mobilisation compared with ACB or ITM alone.

## Data Availability

All relevant data is provided within the manuscript, embedded tables, as well as the uploaded tables (Tables 1, 2, 3). The entire data set is available from the corresponding author on request. All data is stored in a password secured, locked file but can be provided if required by the journal. Please contact the primary author if further data information is required.

## Abbreviations

TKA: Total knee arthroplasty
APS: Acute Pain Service
FMC: Flinders Medical Centre
VAS: Visual Analogue Scale
ITM: Intrathecal Morphine
ACB: Adductor Canal Block
PROSPECT: Procedure-specific postoperative pain management
PONV: Postoperative Nausea and Vomiting
